# Upregulated glycolysis correlates with tumor progression and immune evasion in head and neck squamous cell carcinoma

**DOI:** 10.1038/s41598-021-97292-6

**Published:** 2021-09-07

**Authors:** Hideyuki Takahashi, Reika Kawabata-Iwakawa, Shota Ida, Ikko Mito, Hiroe Tada, Kazuaki Chikamatsu

**Affiliations:** 1 Department of Otolaryngology-Head and Neck Surgery, Gunma University Graduate School of Medicine, 3-39-22 Showa-machi, Maebashi, Gunma 371-8511 Japan; 2 Division of Integrated Oncology Research, Gunma University Initiative for Advanced Research, 3-39-22 Showa-machi, Maebashi, Gunma 371-8511 Japan

**Keywords:** Cancer, Cancer metabolism, Cancer microenvironment, Head and neck cancer, Tumour immunology

## Abstract

Altered metabolism is an emerging hallmark of cancer. Cancer cells preferentially utilize glycolysis for energy production, termed “aerobic glycolysis.” In this study, we performed a comprehensive analysis of the glycolytic activity in head and neck squamous cell carcinoma (HNSCC) using data obtained from The Cancer Genome Atlas database. We first divided 520 patients with HNSCC into four groups based on the mRNA expression of 16 glycolysis-related genes. The upregulated glycolytic activity positively correlated with human papillomavirus-negative tumor type, advanced T factor, and unfavorable prognosis. The gene set enrichment analysis revealed upregulation of several hallmark pathways, including interferon-alpha response, myc targets, unfolded protein response, transforming growth factor-β signaling, cholesterol homeostasis, and interleukin 6-Janus kinase-signal transducer and activator of transcription 3 signaling, in the glycolysis-upregulated groups. Immune cell enrichment analysis revealed decreased infiltration of T cells, dendritic cells, and B cells in the glycolysis-upregulated groups, suggesting impaired tumor antigen presentation, T cell activation, and antibody production in the TME. Moreover, the expression profile of immune-related genes indicated increased immune evasion in the glycolysis-upregulated tumors. Collectively, these findings suggest that transcriptome analysis of glycolytic activity of tumors has the potential as a biomarker for tumor progression and immunological status in patients with HNSCC.

## Introduction

Head and neck squamous cell carcinoma (HNSCC) is the sixth most common cancer worldwide, with a reported incidence of 890,000 new cases and 450,000 deaths annually^1,2^. The major risk factors of HNSCC include exposure to tobacco-derived carcinogens and excessive alcohol consumption^3^. Moreover, prior infection with oncogenic strains of human papillomavirus (HPV) has recently been recognized as a risk factor of oropharyngeal cancers, especially in the USA and Western Europe^4^. As the 5-year survival rate of HNSCC is still 66%, new therapeutic modalities have been explored. Recent breakthroughs in cancer therapy have resulted in novel immunotherapy regimens targeting the immune checkpoint programmed death 1 (PD-1) in patients with HNSCC^5,6^. However, the survival benefit is limited to only 20–30% patients, and new biomarkers that predict the efficacy of anti-PD-1 therapy have been investigated. Accumulating evidence has indicated that in HNSCC, tumor cells orchestrate a highly immunosuppressive tumor microenvironment (TME) via the production of immunosuppressive mediators, recruitment of various stromal cells, expression of immune checkpoint ligands, and downregulation of human leukocyte antigen expression^7,8^, which is referred to as immune evasion^9^. As immune checkpoint inhibitors, including anti-PD-1 antibodies, target the interaction between tumor cells and T cells, a comprehensive understanding of complex immune networks in the TME is needed to establish a new biomarker for immunotherapies.

Reprogramming of energy metabolism is an emerging hallmark of cancers^10,11^. Even during aerobic conditions, cancer cells preferentially utilize glycolysis for producing energy, termed “aerobic glycolysis”^12,13^. Increased glycolysis correlates with aggressive tumor progression, treatment resistance, and unfavorable prognosis in various cancers, including HNSCC^14–17^. The upregulation of glucose transporter-1 (GLUT1), which transports glucose into the cytoplasm, increases glucose utilization and is associated with aggressive behavior in several cancers^18^. Increased glucose uptake via GLUT1 has been investigated in clinics using positron emission tomography, which monitors the uptake of a radiolabeled glucose analog, ^18^F-fluorodeoxyglucose. Moreover, both oncogenic signaling pathways and hypoxia induce aerobic glycolysis in cancer cells. Activation of oncogenes such as *RAS*, *MYC*, and *MAPK* and PI3K-AKT signaling as well as *TP53* mutation, a tumor suppressor gene, are associated with increased glycolysis^19–23^. The hypoxic TME also alters metabolic reactions in tumor cells by upregulating the expression of glucose transporters and enzymes of the glycolysis pathway, including transcription factors, hypoxia-inducible factor (HIF) 1α and HIF2α^24–26^. However, the primary mechanism that regulates glycolysis in cancer cells still remains unclear. Furthermore, the relationship between upregulated glycolysis and clinicopathological features is elusive in various cancer types. Notably, a few studies have revealed the interplay between tumor glycolysis and immune evasion^15,27,28^. We previously reported the positive correlation between glycolytic activity and PD-1-programmed death ligand-1 (PD-L1) expression, which is a ligand of PD-1, in patients with oral squamous cell carcinoma^29^. According to the recent advances in cancer immunotherapies, a comprehensive analysis that focuses on the relationship between glycolysis and immunological significance in the TME is warranted.

In the present study, we performed a comprehensive analysis of glycolysis in HNSCC by analyzing transcriptome and clinical data regarding HNSCC obtained from public database. Based on the mRNA expression of 16 glycolysis-related genes, we segregated patients with HNSCC into groups and performed a comprehensive analysis to compare the clinical and biological significance between the glycolysis groups.

## Results

### Glycolytic activity positively correlated with locally advanced tumors and unfavorable prognosis

**Figure 1:**
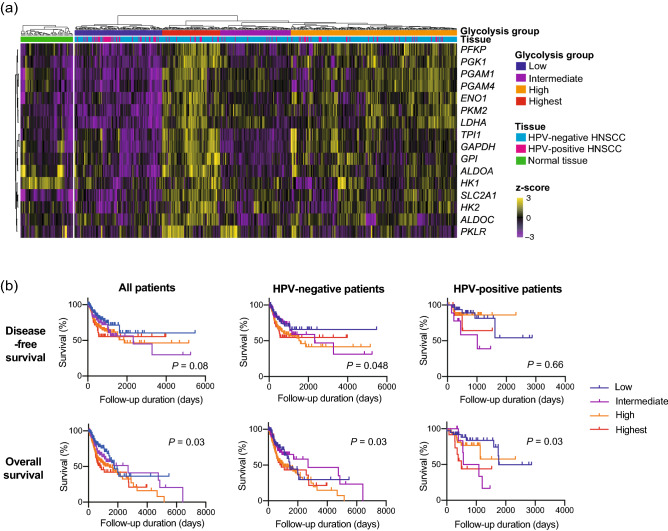
Glycolytic activity positively correlated with unfavorable prognosis. (**a**,**b**) mRNA expression data of 16 glycolysis-related genes and clinical information were obtained from TCGA database. (**a**) Heat map of glycolysis-related gene expression in 44 normal tissues and 520 patients with HNSCC. All patients with HNSCC underwent non-supervised hierarchical clustering based on the z-scores of log_10_-transformed expressions. (**b**) Kaplan–Meier survival curves based on glycolysis groups. Disease-free survival was evaluated in all patients (n = 429), HPV-negative patients (n = 348), and HPV-positive patients (n = 81). Overall survival was evaluated in all patients (n = 495), HPV-negative patients (n = 403), and HPV-positive patients (n = 92). TCGA, The Cancer Genome Atlas; HNSCC, head and neck squamous cell carcinoma; HPV, human papillomavirus.

We first performed non-supervised hierarchical clustering of 520 patients with HNSCC based on data on the expression of 16 glycolysis-related genes obtained from The Cancer Genome Atlas (TCGA) database (Fig. 1a). Then, patients were divided into four glycolysis groups, including low, intermediate, high, and highest. The expression of most glycolysis-related genes was higher in the glycolysis-high and glycolysis-highest group than that in normal tissues, except *PGK1*, *ALDOA* and *HK1* (Suppl. Figure 1). The glycolysis groups were compared in terms of clinical parameters, including HPV status, primary lesion, T factor, N factor, M factor, and tumor-node-metastasis (TNM) stage (Table 1). The proportion of HPV-positive patients in the glycolysis-low group was significantly higher (35.2%) than those in the other groups. Regarding primary lesions, the proportion of oral cavity lesions was lower (55.5%), whereas the proportion of oropharynx lesion was higher (17.6%) in the glycolysis-low group than those in the other groups. In the glycolysis-low group, the proportion of patients with T0, T1, and T2 tumors (51.3%) was higher than that of patients with T3 and T4 tumors (48.7%). However, in the other groups, the proportion of patients with T0, T1, and T2 tumors was lower than that of patients with T3 and T4 tumors. No differences in the N factor, M factor, and TNM stage were observed between the groups. Alternatively, we performed hierarchical clustering of 270 patients with HNSCC obtained from Gene Expression Omnibus (GEO) database (Suppl. Figure 2a). Although not significant, similar trends were observed in the GSE65858 cohort (Suppl. Table 1). Additionally, we performed survival analyses to compare survivals between the groups (Fig. 1b, Suppl. Figure 2b). In the TCGA cohort, the upregulated glycolysis correlated with shorter overall survival (OS) in all patients. In HPV-negative patients, the upregulated glycolysis correlated with both shorter disease-free survival (DFS) and shorter OS. In HPV-positive patients, although the significant difference of survival curves between the glycolysis groups was observed, no linear trend between upregulated glycolysis and OS was identified. In the GSE65858 cohort, no correlation was observed between survivals and glycolysis groups in all patients and HPV-negative patients. Meanwhile, the upregulated glycolysis correlated with both shorter DFS and OS in HPV-positive patients.

**Table 1:** Relationship between glycolytic activity and clinical parameters in 520 patients with HNSCC.

Variables	Glycolysis				
	Low (n = 119)	Inter (n = 96)	High (n = 226)	Highest (n = 79)	*P*-value
**HPV status**					
Negative	77	87	192	67	< 0.0001
Positive	42	9	34	12	
**Primary lesion**					
Hypopharynx	2	0	7	1	0.0003
Larynx	30	16	49	21	
Oral cavity	66	79	154	52	
Oropharynx	21	1	16	5	
**T factor**					
T0-2	61	42	79	28	0.02
T3-4	58	54	147	51	
**N factor**					
Negative	41	46	94	32	0.24
Positive	70	44	119	45	
**M factor**					
Negative	116	94	219	75	0.36
Positive	0	1	2	2	
**TNM stage**					
I–II	25	26	47	13	0.38
III–IV	94	70	179	66	

HNSCC, head and neck squamous cell carcinoma; HPV, human papillomavirus; TNM, tumor-node-metastasis; inter, intermediate.

### Glycolytic activity positively correlated with several pro-tumoral pathways

**Figure 2:**
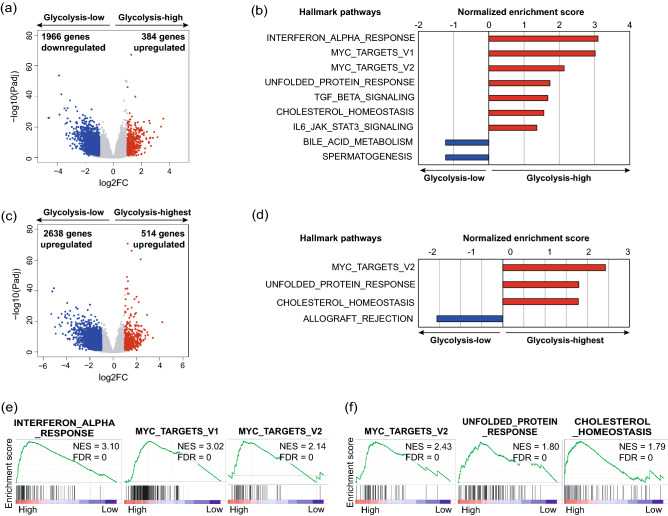
Several pro-tumoral pathways were upregulated in the glycolysis-upregulated groups. (**a**–**f**) mRNA expression data were obtained from TCGA database. (**a**,**c**) Volcano plot of differentially expressed genes in the glycolysis-high group or the glycolysis-highest group compared to the glycolysis-low group. Red dots represent upregulated genes (Padj < 0.05, log2FC > 1), whereas blue dots represent downregulated genes (Padj < 0.05, log2FC < -1). (**b**) Upregulated and downregulated hallmark pathways in the glycolysis-high group compared to the glycolysis-low group obtained by GSEA (FDR < 0.10). (**d**) Upregulated and downregulated hallmark pathways in the glycolysis-highest group compared to the glycolysis-low group obtained by GSEA (FDR < 0.10). (**e**) Representative GSEA plots of upregulated pathways shown in (**b**). (**f**), Representative GSEA plots of upregulated pathways shown in (**d**). TCGA, The Cancer Genome Atlas; Padj, adjusted *P-value*; GSEA, gene set enrichment analysis.

To investigate the biological significances in the glycolysis-upregulated groups, we compared the glycolysis groups in the TCGA cohort and identified 2350 differentially expressed genes (DEGs), including 384 upregulated and 1966 downregulated genes in the glycolysis-high group compared to the glycolysis-low group (Fig. 2a, Suppl. Table 2). We also identified 3152 DEGs, including 514 upregulated and 2638 downregulated genes in the glycolysis-highest group compared to the glycolysis-low group (Fig. 2c, Suppl. Table 3). To explore the biological functions and related pathways upregulated in the glycolysis-high group and the glycolysis-highest group, we conducted gene set enrichment analysis (GSEA; Fig. 2b,d,e,f). In the glycolysis-high group, seven hallmark pathways were upregulated and two pathways were downregulated (FDR < 0.10). Several pathways representing cancer hallmarks, such as interferon-alpha (IFN-α) response, myc targets, unfolded protein response, transforming growth factor (TGF)-β signaling, cholesterol homeostasis, and interleukin 6 (IL6)-Janus kinase (JAK)-signal transducer and activator of transcription 3 (STAT3) signaling, were upregulated in the glycolysis-high group. In the glycolysis-highest group, three hallmark pathways were upregulated and one pathway was downregulated (FDR < 0.10). The pathways representing cancer hallmarks, including myc targets, unfolded protein response, and cholesterol homeostasis, were upregulated in the glycolysis-highest group. We alternatively performed GSEA using the GSE65858 cohort (Suppl. Figure 3). In the glycolysis-high group, 19 hallmark pathways representing cancer hallmarks were upregulated and 5 pathways representing inflammatory response were downregulated (FDR < 0.05). The upregulated and downregulated pathways were consistent with those in the TCGA cohort.

### Glycolytic activity negatively correlated with immune cell enrichments that facilitate anti-tumor immunity

**Figure 3:**
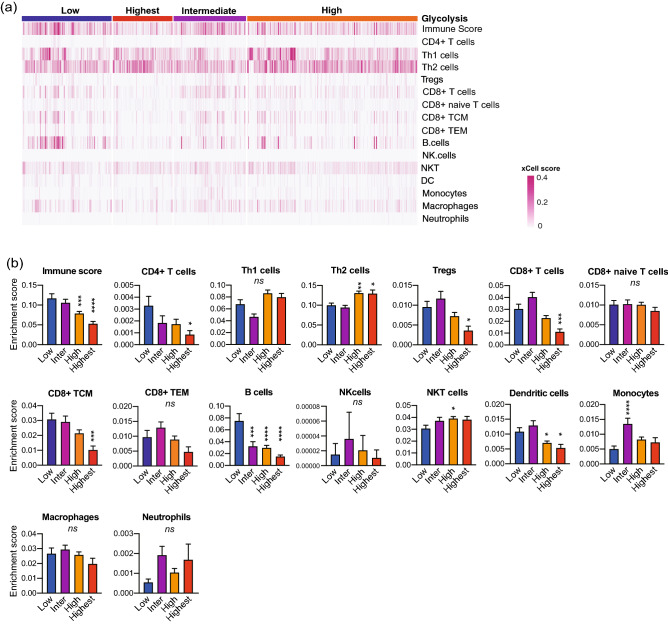
Glycolytic activity negatively correlated with immune cell enrichments that facilitate anti-tumor immunity. (**a**) Heat map of immune cell enrichment scores in 520 patients with HNSCC obtained from TCGA database. The enrichment scores of basic immune cell types were calculated using the xCell tool. (**b**) Bar graphs of immune cell enrichment scores in the glycolysis groups shown in a. The enrichment scores in the glycolysis-intermediate, the glycolysis-high, and the glycolysis-highest group were compared to those in the glycolysis-low group. *, *P* < 0.05; **, *P* < 0.01; ***, *P* < 0.001; ****, *P* < 0.0001. HNSCC, head neck squamous cell carcinoma; TCGA, The Cancer Genome Atlas; TCM, central memory T cells; TEM, effector memory T cells.

We next calculated the enrichment scores of basic immune cell types to assess the correlation between glycolytic activity and immune cell infiltration in the TCGA cohort (Fig. 3). In the glycolysis-high group, the immune score and the enrichment scores of B cells and dendritic cells (DCs) were lower than those in the glycolysis-low group. Meanwhile, the enrichment score of Th2 cells was higher in the glycolysis-high group than that in the glycolysis-low group. In the glycolysis-highest group, the immune score and the enrichment scores of CD4 + T cells, CD8 + T cells, CD8 + central memory T cells (TCM), regulatory T cells (Tregs), B cells, and DCs were lower than those in the glycolysis-low group. Meanwhile, the enrichment score of Th2 cells was higher in the glycolysis-highest group than that in the glycolysis-low group. The enrichment scores of immune cells were also calculated in the GSE65858 cohort, indicating the similar trends with the TCGA cohort except NKT cells (Suppl. Figure 4).

### The expression profile of immune-related genes indicated increased immune evasion in glycolysis-upregulated tumor microenvironment

**Figure 4:**
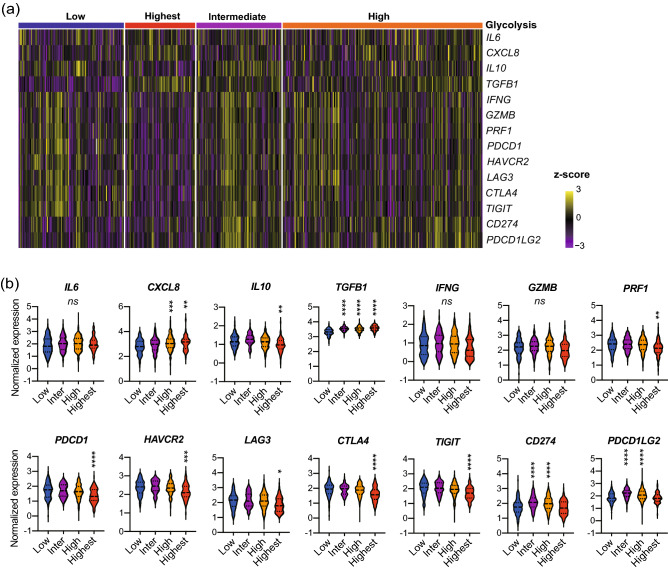
The expression profile of immune-related genes indicated increased immune evasion in glycolysis-upregulated tumors. (**a**) Heat map of immune-related gene expression in 520 patients with HNSCC obtained from TCGA database. (**b**) Violin plots of normalized gene expressions in the glycolysis groups shown in (**a**). The gene expressions in the glycolysis-intermediate, the glycolysis-high, and the glycolysis-highest group were compared to those in the glycolysis-low group.*, *P* < 0.05; **, *P* < 0.01; ***, *P* < 0.001; ****, *P* < 0.0001. HNSCC, head neck squamous cell carcinoma; TCGA, The Cancer Genome Atlas.

We compared the expression of immune-related genes to emphasize immunological differences between the glycolysis groups (Fig. 4). In the glycolysis-high group, the expression of *TGFB1*, *CXCL8*, *CD274*, and *PDCD1LG2* was higher than that in the glycolysis-low group. In the glycolysis-highest group, the expression of *TGFB1* and *CXCL8* was higher, whereas that of *LAG3*, *PRF1*, *HAVCR2*, *CTLA4*, *PDCD1*, *TIGIT*, and *IL10* was lower than that in the glycolysis-low group. The gene expressions were also compared to the glycolysis groups in the GSE65858 cohort (Suppl. Figure 5). Similar trends were observed in *CXCL8*, *IL10*, *PRF1*, *PDCD1*, *HAVCR2*, *LAG3*, *CTLA4*, and *TIGIT*. In addition, the expression of *IFNG* and *GZMB* was lower in the glycolysis-high group than that in the glycolysis-low group.

## Discussion

Altered metabolism is an emerging hallmark of cancer^10,11^. Various cancers exhibit upregulated glycolysis and downregulated mitochondrial oxidative phosphorylation. Meanwhile, immune evasion has recently attracted attention due to recent advances in cancer immunotherapy. In the present study, we performed a comprehensive analysis of the transcriptome and clinical data regarding HNSCC obtained from TCGA database. The glycolytic activity positively correlated with HPV-negative tumor type, advanced T factor, and unfavorable prognosis. GSEA revealed that the glycolytic activity positively correlated with the upregulation of various pathways representing cancer hallmarks. The immune cell enrichment analyses showed the negative correlation between the glycolytic activity and the infiltration of T cells, DCs, and B cells. Moreover, the expression profile of immune-related genes indicated increased immune evasion in the glycolysis-upregulated tumors. These results suggest that transcriptome analysis of glycolytic activity of tumors has the potential as a biomarker for tumor progression and immunological status in patients with HNSCC.

Non-supervised hierarchical clustering analysis revealed a positive correlation between glycolysis and advanced T factors in the TCGA cohort. In normal cells, mitochondrial oxidative phosphorylation is widely used to efficiently generate energy. However, cancer cells often rely on aerobic glycolysis, resulting in remarkably upregulated glycolysis, although not an efficient strategy to produce adenosine 5’-triphosphate (ATP). Accelerated cell proliferation has been implicated in the dependence of cancer cells on glycolysis. For rapid proliferation, cells require nutrient metabolism to generate nucleotides, amino acids, and lipids rather than efficient ATP production. In cancer cells, upregulated glycolysis results from altered metabolic pathways that provide biomass for cell proliferation^12,30^. Consequently, the advanced T factor in the glycolysis-upregulated groups could be attributed to accelerated cell proliferation, which relies on aerobic glycolysis. The upregulated unfold protein response pathway and cholesterol homeostasis pathway in the glycolysis-upregulated groups obtained by GSEA could also be related to accelerated cell proliferation. Furthermore, we observed a positive correlation between the HPV-negative tumor type and the glycolytic activity. Comprehensive analysis of the TCGA database indicated frequent alterations in *TP53* and *CDKN2A* in HPV-negative HNSCC, but not in HPV-positive HNSCC^31^. *TP53* is a negative regulator of glycolysis^32^. Moreover, in vitro experiment using both HPV-negative and HPV-positive cell lines indicated that HPV-negative cells have higher glycolytic activity than HPV-positive cells^33^. Collectively, upregulated glycolysis may be a biological feature of HPV-negative carcinogenesis. In addition, upregulated glycolysis was an unfavorable prognostic factor. Upregulation of glycolysis in cancer cells results in the accumulation of lactate in the TME^12^. A high lactic acid concentration in the TME inhibits the cytotoxic activity of cytotoxic T cells, resulting in immune evasion of tumors^34^. A lactate-enriched TME also mediates increased invasion, apoptosis resistance, and increased survival and proliferation of tumor cells^35–37^. Therefore, advanced T factor and a lactate-enriched TME may be associated with shorter survivals in the glycolysis-upregulated patients.

GSEA revealed that various pathways that represent cancer hallmarks were upregulated in the glycolysis-upregulated tumors. The IFN-α response pathway exhibited the highest normalized enrichment score in the glycolysis-high group. IFN-α is a cytokine family that belongs to type I IFNs^38^. Based on its stimulatory effects on innate and adaptive immunity, IFN-α has been widely used in clinical oncology to treat various cancer types, especially hematological malignancies^39^. However, recent studies have demonstrated the pro-tumoral role of IFN-α signaling. In HNSCC, IFN-α signaling promotes the immunosuppressive TME by activating the PD-1-programmed death ligand-1 (PD-L1) axis^40^. Therefore, the upregulation of PD-1 ligand genes, *CD274* and *PDCD1LG2*, in the glycolysis-high group could be related to the upregulated IFN-α response. Although the abundance of IFN-γ in the TME has been widely recognized as a key mechanism that induces PD-L1 expression in tumor cells^41^, our results suggest that IFN-α could also be an important mechanism for PD-L1 induction in glycolysis-upregulated HNSCCs. Moreover, growing evidence suggests that IFN-α signaling contributes to increased migration, drug resistance, and immune evasion in breast cancers^42^. Further investigations focused on the relationship between IFN-α signaling and glycolysis in the TME of HNSCC are needed. Furthermore, GSEA revealed that that the TGF-β signaling pathway was upregulated in the glycolysis-high group. TGF-β is a well-known cytokine family and a major driver of epithelial-mesenchymal transition (EMT)^43^. Accumulating evidence suggests that TGF-β signaling also enhances glycolysis by activating various glycolytic enzymes, including GLUT1, hexokinase 2, 6-phosphofructo-2-kinase/fructose-2,6-biphosphatase 3, 6-phosphofructo-1-kinase, pyruvate kinase M2, and lactate dehydrogenase type A^44^. Consistent with these findings, TGF-β signaling may play a vital role in the induction of both glycolysis and EMT in HNSCC. Additionally, the significant upregulation of myc target pathways was consistent with their roles in the induction of glycolysis^20,24–26^.

Immune cell enrichment analysis revealed a negative correlation between the upregulated glycolysis and infiltration of CD4 + T cells, CD8 + T cells, and DCs. The interaction between DCs and T cells is an important axis of the anti-tumor immune response through tumor-antigen presentation and T cell activation^45^. The negative effects of the lactate-enriched TME on T cell proliferation, cytotoxic activity of T cells, and maturation of DCs support our observation^46,47^. Moreover, the comparison of immune-related gene expression between the glycolysis groups revealed higher expressions of *CD274*, *PDCD1LG2*, and *TGFB1* in the glycolysis-high group, which facilitated T cell exhaustion and dysfunction in the TME^48^. Therefore, the decreased infiltration of T cells and DCs in the glycolysis-high group may be related to increased immune evasion in the glycolysis-upregulated tumors. Furthermore, the downregulated expression of *PRF1* and immune checkpoint molecules, including *LAG3*, *HAVCR2*, *CTLA4*, *PDCD1*, and *TIGIT*, was observed in the glycolysis-highest group. As these immune checkpoint molecules function as receptors for T cell inactivation and exhaustion signals, these molecules are abundantly expressed on effector memory T cells and tissue-resident memory T cells, which are activated phenotypes of T cells ^49–52^. The downregulation of these genes may also be an aspect of the increased immune evasion in the TME. We also observed a negative correlation between glycolysis and enrichment of B cells. Recent advances in cancer immunotherapy targeting solid tumors have mainly focused on T cells; however, the anti-tumor roles of B cells through antigen presentation and antibody production have been recently recognized^53^. The significantly decreased B cell enrichment in the glycolysis-upregulated tumors may also reflect immune evasion in the TME of HNSCC. We observed the lower enrichment of Tregs in the glycolysis-highest group than in the glycolysis-low group. Tregs are known to be an immunosuppressive subset of CD4 + T cells; however, their functions and prognostic values differ in their location and inflammatory state in the TME of HNSCC^54^. The relationship between glycolytic status and Treg enrichment needs to be further investigated regarding these aspects.

There are limitations to the present study. We performed a comprehensive analysis using bulk RNA sequencing data. In the case of bulk RNA sequencing, gene expression values represent not only mRNA expression in tumor cells but also that in all other cell types existing in the TME. This method should work well for genes that are specific to certain cell types; however, for genes that are commonly expressed in various cell types, single-cell RNA sequencing would be better suited for a more precise analysis. Accumulation of public archives of single-cell RNA sequencing data is warranted for an in-depth characterization of the metabolic state of tumors.

In conclusion, we elucidated the correlation between glycolytic activity and several clinical and transcriptomic significances in HNSCC by analyzing data obtained from public database. Transcriptome analysis of glycolytic activity of tumors has the potential as a biomarker for tumor progression and immunological status in patients with HNSCC.

## Materials and Methods

### Acquisition of public database

RNA-sequencing data (Illumina Hiseq RNAseq V2, raw counts and normalized counts) and clinical information were obtained from TCGA Research Network (TCGA Provisional version updated in 2016, http://cancergenome.nih.gov/). Data on 564 samples, including 44 normal tissues, 97 patients with HPV-positive HNSCC, and 423 patients with HPV-negative HNSCC, were analyzed. The log_10_-transformed values of mRNA expression levels were calculated. Alternatively, GSE65858 dataset, including microarray data (Illumina HumanHT-12 V4.0 expression beadchip platform) and clinical data, were obtained from GEO database. A total of 270 cases, consisting of 73 HPV-positive HNSCCs, 196 HPV-negative HNSCCs, and 1 HPV-unknown HNSCC were analyzed.

### Hierarchical clustering of patients with glycolysis-related genes

All patients with HNSCC underwent non-supervised hierarchical clustering based on z-scores of the expression of glycolysis-related genes. The 16 glycolysis-related genes specifically expressed in several cancer types were chosen from glycolysis pathway of the Reactome pathway database: *PFKP*, *PGK1*, *PGAM1*, *PGAM4*, *ENO1*, *PKM2*, *LDHA*, *TPI1*, *GAPDH*, *GPI*, *ALDOA*, *HK1*, *SLC2A1*, *HK2*, *ALDOC*, and *PKLR* based on the previous reports ^55–58^. Patients in the TCGA cohort were divided into four groups—low, intermediate, high, and highest—using the cutree R function based on the clustering result. The number of clusters was set as four for dividing clusters. The expression of 16 glycolysis-related genes in the glycolysis groups was compared to that in normal tissues. The heat map was illustrated using the pheatmap R package. Alternatively, patients in the GSE65858 cohort were divided into three groups—low, intermediate, and high—using the cutree R function based on the clustering result. The number of clusters was set as three for dividing clusters.

### Differentially expressed gene analysis

DEGs were identified using the ExperimentHub R package and DESeq2 R package. DEGs were filtered using the threshold |log_2_FC|≥ 1 and adjusted *P*-value of < 0.05. A volcano plot was used to visualize DEGs using the calibration R package.

### Gene set enrichment analysis

GSEA (GSEA v4, Broad Institute) was performed to identify upregulated and downregulated pathways in the glycolysis-upregulated groups using normalized counts of all genes. The normalized enrichment score, *P*-value, and false discovery rate (FDR) q-value were calculated for each gene set using the Hallmark pathway database.

### Cell type enrichment analysis

The xCell tool, a gene signature-based method, was employed to evaluate the enrichment of various cell types in HNSCC tissues. The normalized counts of all genes were used as input. Among the 64 cell types, the enrichment scores of basic immune cell types were calculated using the xCell R package.

### Statistical analysis

Data were analyzed using R (version 4.0.3; The R Foundation for Statistical Computing, Vienna, Austria) in combination with R studio version 1.3.1093 (R studio, Boston, MA, USA) and GraphPad Prism version 8 (GraphPad Software, San Diego, CA, USA). One-way ANOVA with Tukey’s post-hoc test for multiple pairwise testing was used to compare continuous variables between the glycolysis groups. The Chi-square test for independence was used to compare categorical variables. Two-sided *P*-values of < 0.05 were considered statistically significant. Survival curves were calculated using the Kaplan–Meier method and compared using the log-rank test. In the TCGA cohort, DFS was evaluated in all patients (n = 429), HPV-negative patients (n = 348), and HPV-positive patients (n = 81); whereas OS was evaluated in all patients (n = 495), HPV-negative patients (n = 403), and HPV-positive patients (n = 92). In the GSE65858 cohort, both DFS and OS were evaluated in all 270 patients.

## Supplementary Information

Supplementary Figures.

Supplementary Tables.
