# Supplementary material for: Upregulated glycolysis correlates with tumor progression and immune evasion in head and neck squamous cell carcinoma
Source: Sci Rep. 2021 Sep 7;11:17789. doi: 10.1038/s41598-021-97292-6 (PMC8423753; doi:10.1038/s41598-021-97292-6)
Supplement: Supplementary file 1 — Supplementary Figures. [file 41598_2021_97292_MOESM1_ESM.pdf]

Suppl.Figure 1

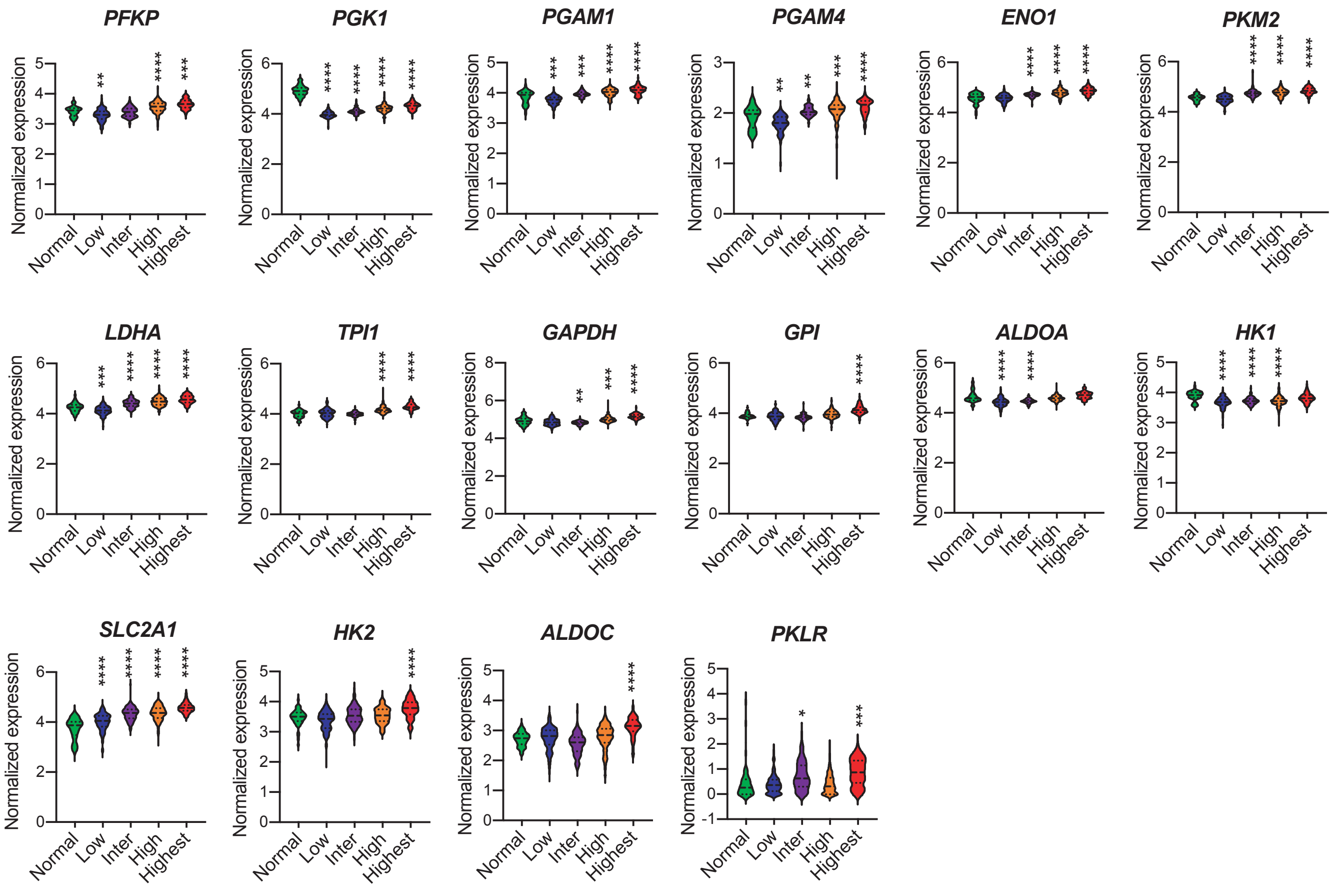

Suppl.Figure 1. The expression of 16 glycolysis-related genes in normal tissues and HNSCCs.

Violin plots showing normalized gene expressions of 16 glycolysis-related genes in glycolysis groups of 520 HNSCC samples and 44 normal tissues obtained from TCGA database. The gene expressions in glycolysis groups were compared to those in normal tissues. *, $P < 0.05$; **, $P < 0.01$; ***, $P < 0.001$; ****, $P < 0.0001$. HNSCC, head neck squamous cell carcinoma, TCGA, The Cancer Genome Atlas.

Suppl.Figure 2

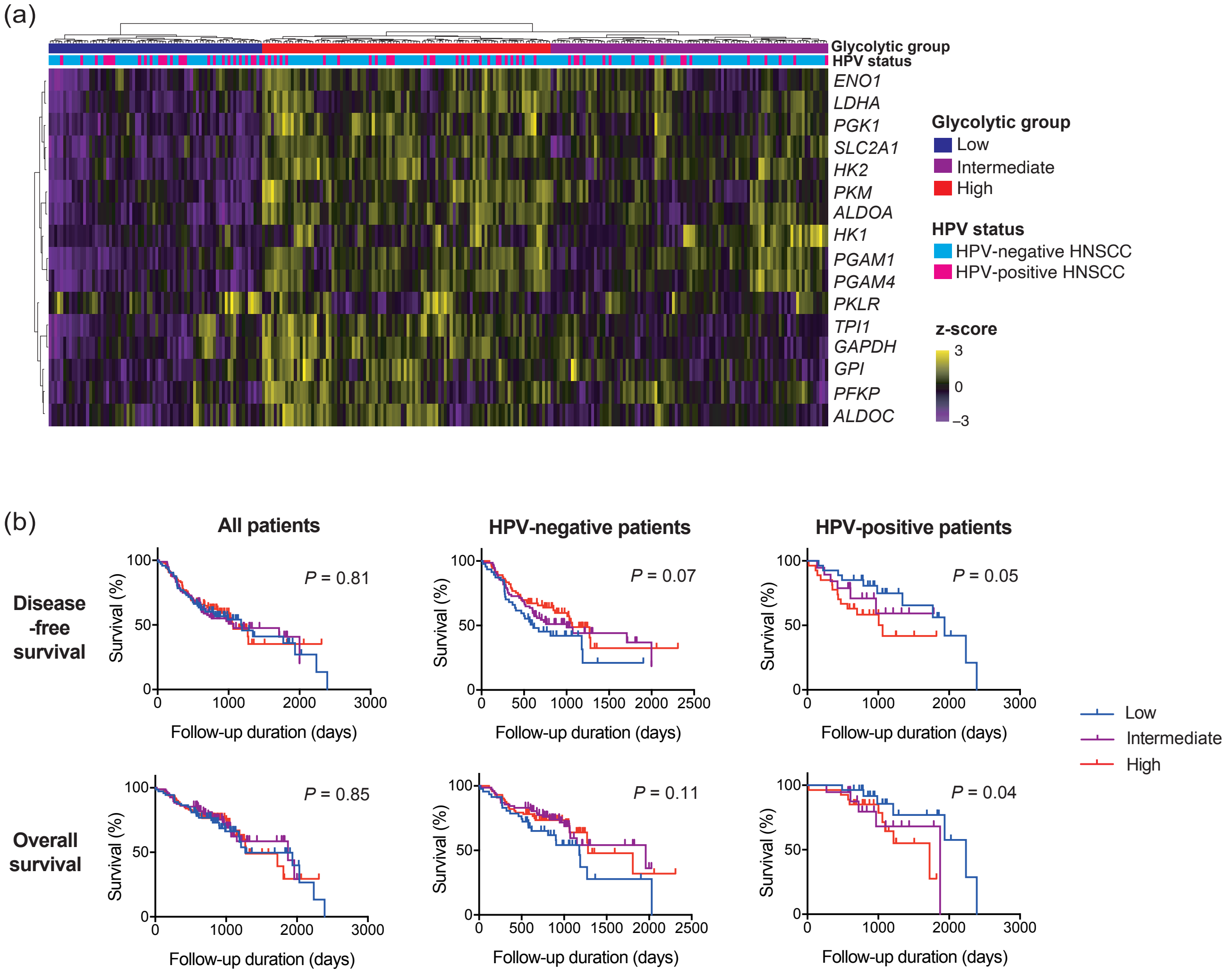

Suppl.Figure 2. Glycolytic activity correlated with unfavorable prognosis in patients with HPV-positive HNSCCs

a-b, mRNA expression data of 16 glycolysis-related genes and clinical information were obtained from GEO database. a, Heat map of glycolysis-related gene expression in 270 patients with HNSCC. Patients underwent non-supervised hierarchical clustering based on the z-scores of log10-transformed expressions. b, Kaplan-Meier survival curves based on glycolysis groups. Disease-free survival and overall survival were evaluated in all patients (n = 270), HPV-negative patients (n = 196), and HPV-positive patients (n = 73), respectively. GEO, Gene Expression Omnibus; HNSCC, head and neck squamous cell carcinoma; HPV, human papillomavirus.

Suppl.Figure 3

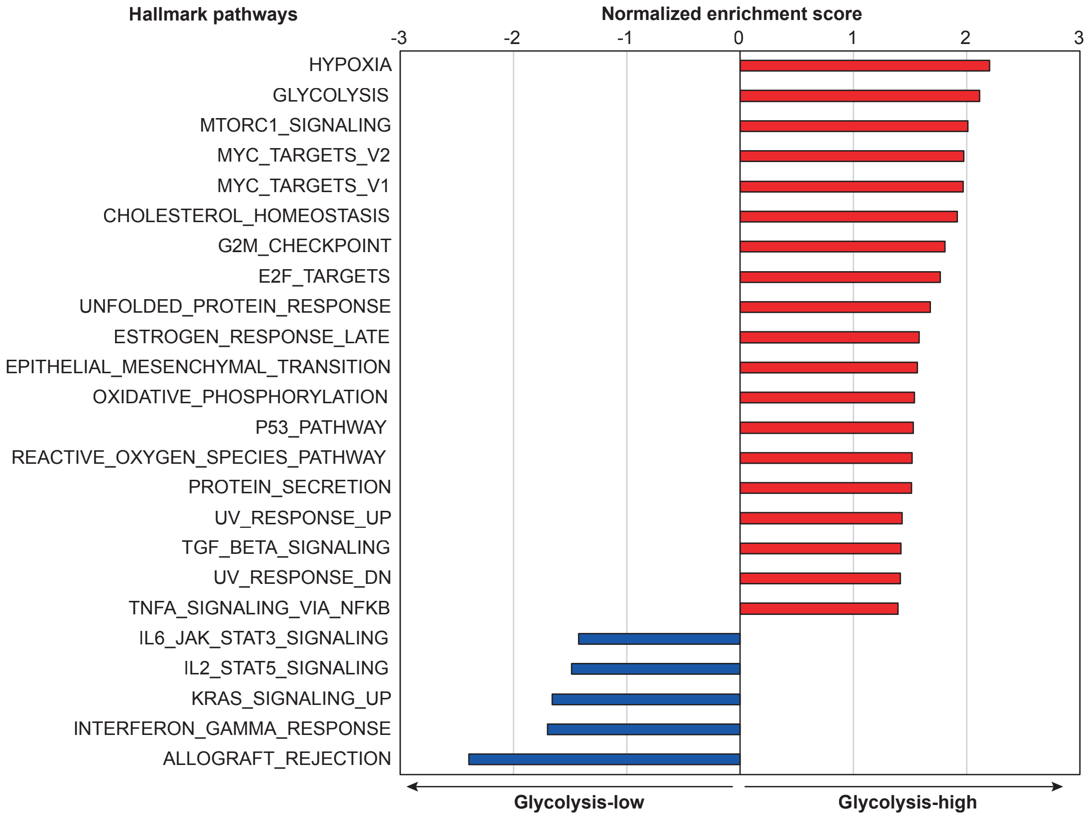

Suppl.Figure 3. Several pro-tumoral pathways were upregulated in the glycolysis-high group

mRNA expression data of patients with HNSCC were obtained from GEO database. Upregulated and downregulated hallmark pathways in the glycolysis-high group compared to the glycolysis-low group calculated by GSEA (FDR < 0.05) are shown. HNSCC, head and neck squamous cell carcinoma; GEO, Gene Expression Omnibus; GSEA, gene set enrichment analysis.

Suppl. Figure 4

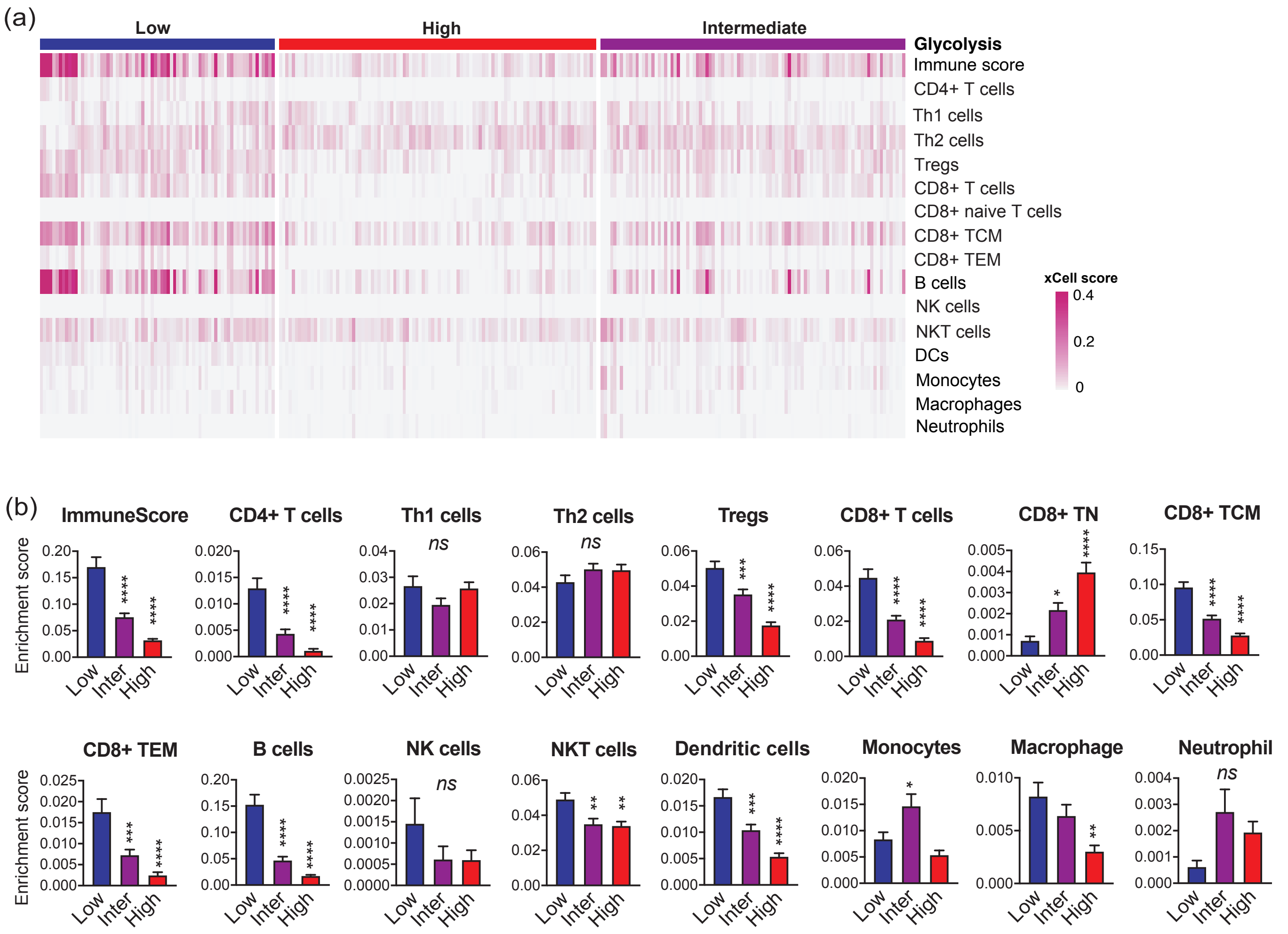

Suppl.Figure 4. Glycolytic activity negatively correlated with immune cell enrichments that facilitate anti-tumor immunity

a, Heat map of immune cell enrichment scores in 270 patients with HNSCC obtained from GEO database. The enrichment scores of basic immune cell types were calculated using the xCell tool. b, Bar graphs of immune cell enrichment scores in the glycolysis groups shown in a. The enrichment scores in the glycolysis-intermediate and the glycolysis-high group were compared to those in the glycolysis-low group. *, $P < 0.05$; **, $P < 0.01$; ***, $P < 0.001$; ****, $P < 0.0001$. HNSCC, head neck squamous cell carcinoma; GEO, Gene Expression Omnibus.

Suppl.Figure 5

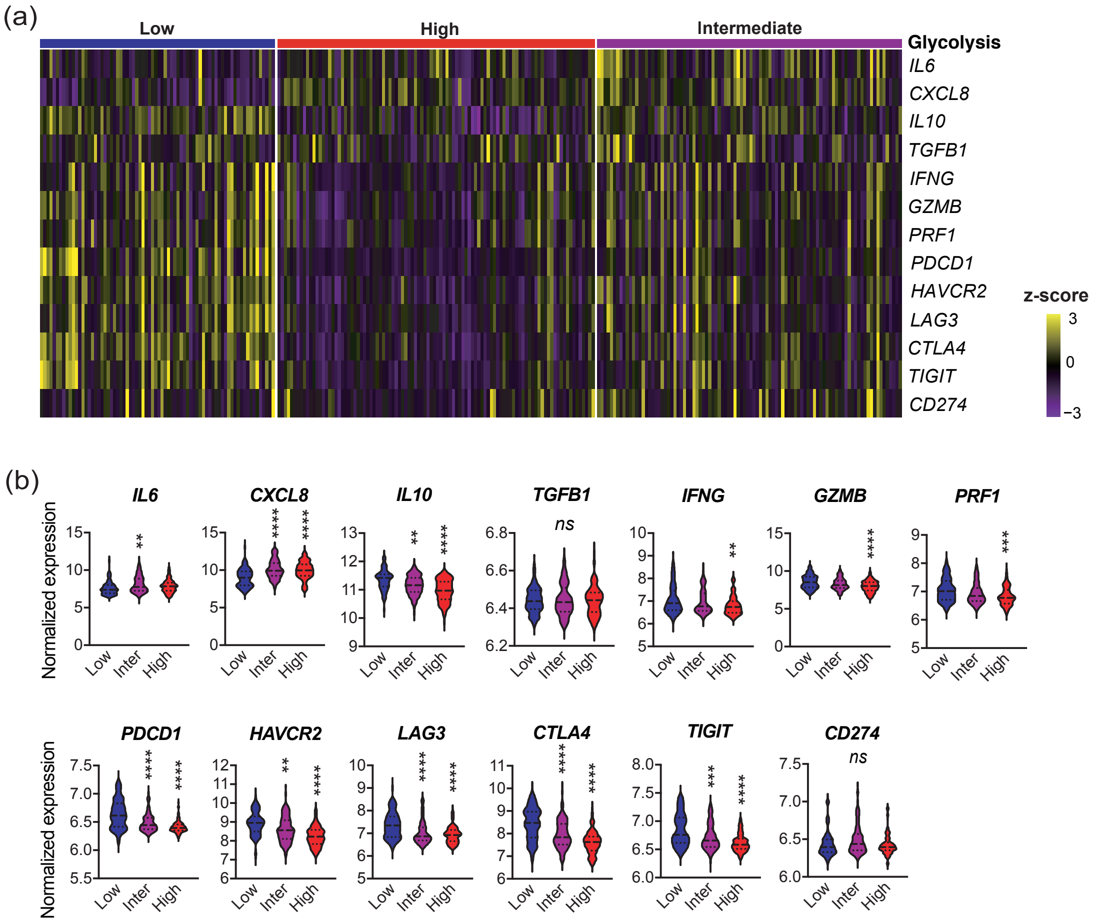

Suppl.Figure 5. The expression profile of immune-related genes indicated increased immune evasion in glycolysis-upregulated tumor microenvironment

a, Heat map of immune-related gene expression in 270 patients with HNSCC obtained from GEO database. b, Violin plots of normalized gene expressions in the glycolysis groups shown in a. The gene expressions in the glycolysis-intermediate and the glycolysis-high group were compared to those in the glycolysis-low group.*, $P < 0.05$; **, $P < 0.01$; ***, $P < 0.001$; ****, $P < 0.0001$. HNSCC, head neck squamous cell carcinoma; GEO, Gene Expression Omnibus.
